# Remote neurostimulation with physical fields at cellular level enabled by nanomaterials: Toward medical applications

**DOI:** 10.1063/5.0022206

**Published:** 2020-11-05

**Authors:** Zixing Xu, Jinhua Xu, Wenjuan Yang, Huoyue Lin, Gang Ruan

**Affiliations:** 1 Department of Biomedical Engineering, College of Engineering and Applied Sciences, Nanjing University, Nanjing City 210093, China; 2 Institute of Materials Engineering, Nanjing University, Nanjing City 210093, China; 3 Jiangsu Key Laboratory of Artificial Functional Materials, Nanjing University, Nanjing City 210093, China

## Abstract

Most neurological diseases have no cure today; innovations in neurotechnology are in urgent need. Nanomaterial-based remote neurostimulation with physical fields (NNSPs) is an emerging class of neurotechnologies that has generated tremendous interest in recent years. This perspective focuses on the clinical translation of this new class of neurotechnologies, an issue that so far has not received enough attention. We outline the major barriers in their clinical translation. We highlight our recent efforts to tackle these translational barriers, with a focus on the biological delivery problem. In particular, for the first time, we have shown that it is feasible to use noninvasive brain delivery to generate significant physiological responses in living animals by NNSP. However, much more work is needed to overcome the translational barriers.

## I. INTRODUCTION

Nanomaterials hold a paradigm-shifting potential for neurotechnology. Published in 2014 by BRAIN Initiative of the U.S., ‘BRAIN 2025: a Scientific Vision’ viewed the potential of nanomaterials as being “revolutionary,” but only recognized their applications as passive probes for neural activities.^1^ The applications of nanomaterials to activate and modulate neural activities were neglected. In 2015, Polina Anikeeva's research group published the first successful nanomaterial-based remote neurostimulation with physical fields (NNSP) in live animals.^2^ This work generated considerable interest and imaginations from the scientific community. In addition to being used as neuroscience tools, it can also be envisioned to employ this new class of neurotechnologies for neuromedicine, to tackle neurological disorders such as Alzheimer's disease, Parkinson's disease (PD), depression, stroke, and epilepsy. However, for this possibility to become a reality, several formidable hurdles in the clinical translation need to be overcome. In this Perspective, we will first briefly review the somewhat surprisingly long (∼20 years) history of NNSP. This will be followed by a discussion of the major challenges in translating this new class of technologies to the medical arena. We will then highlight our recent efforts to address these challenges.

In the literature, there are existing comprehensive reviews on the following topics: (1) various methods of neural stimulation and modulation without the use of nanomaterials,^3,4^ (2) various types of nanomaterials for biomedicine,^5,6^ and (3) various ways of using nanomaterials in neural stimulation and modulation, including but not limited to NNSP.^7–9^ Here, this perspective will focus on the authors' viewpoints and the most relevant studies in the literature. The readers who are interested in more comprehensive information about the above topics are referred to the above-cited references.

## II. NNSP

Combined with proper instrumentation to generate physical fields (e.g., magnetic, electric, and optical fields), nanomaterials offer the following capabilities for neurostimulation and neuromodulation usually not available from conventional techniques such as deep brain stimulation: (1) interfacing with the nervous system at cellular and molecular levels. This is due to the nanometer scale interface provided by nanomaterials. (2) Remote and wireless control by a wide variety of mechanisms. This is owing to the numerous extraordinary magnetic, electrical, optical, and thermal functions of various nanomaterials.^10–12^

**FIG. 1.**
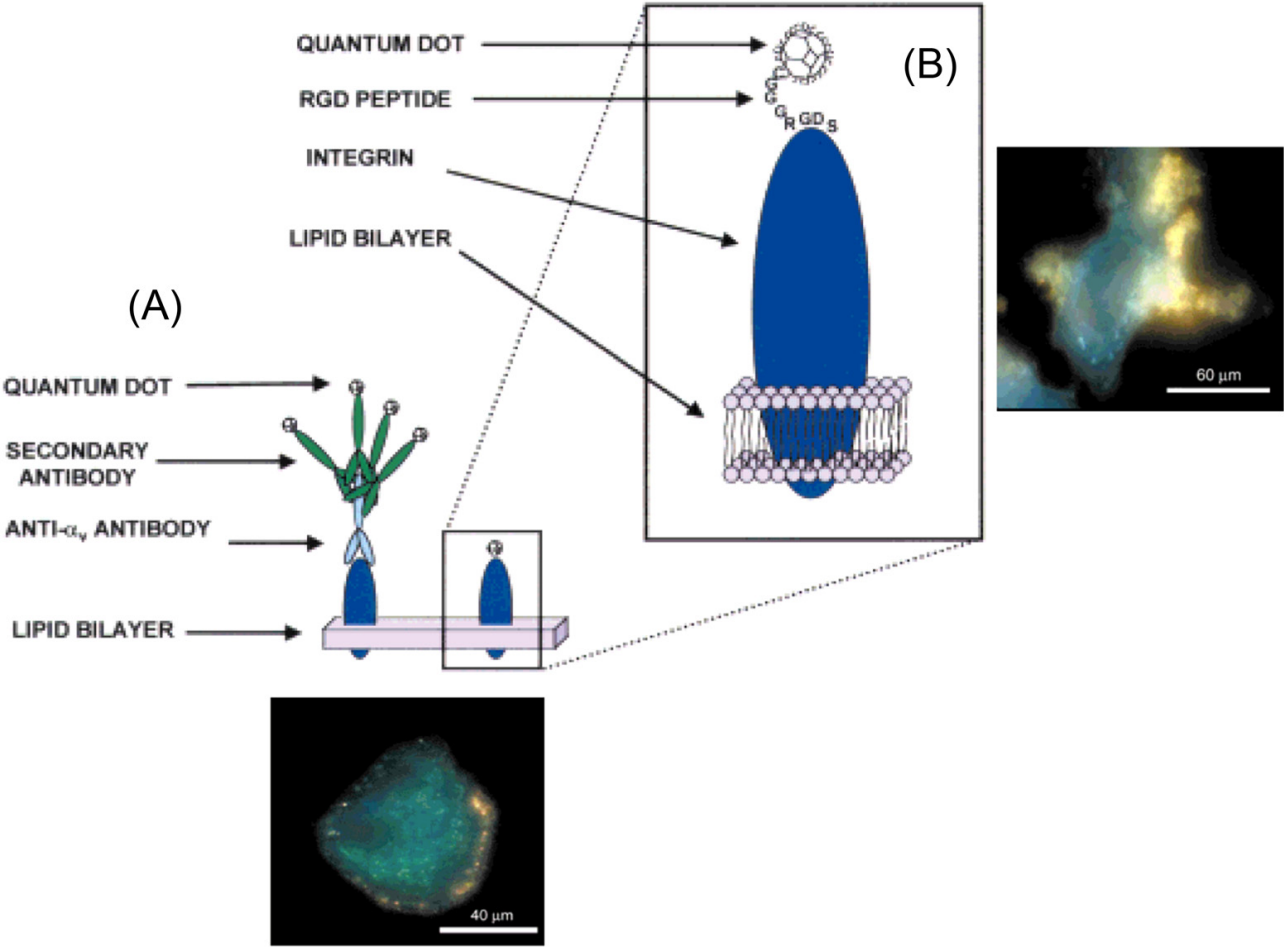
First attempt of NNSP *in vitro*. In this work, quantum dots were conjugated to an antibody (a) or a peptide (b), to specifically recognize integrin receptors on neurons. An optical field was used to remotely stimulate quantum dots-attached neurons. Fluorescent microscopy images of neurons labeled by these two different molecules are shown at the bottom and on the right, respectively. Yellow color is from quantum dots; blue color is from autofluorescence of neurons. Adapted with permission from Winter *et al.*, Adv. Mater. **13**, 1673–1677 (2001). Copy right 2001 John Wiley and Sons.

In a report published in 2001, Jessica Winter, then a graduate student under the joint guidance of Christine Schmidt and Brian Korgel, conducted the first attempt of NNSP.^13^ Semiconductor nanocrystals, or commonly known as quantum dots, were conjugated with an antibody or a peptide to specifically recognize integrin receptors on the cell surface of neurons (Fig. 1). The intended neurostimulation was through using neuron-attached quantum dots to absorb photons (optical field), to convert them into electrons, and thus to activate neurons. However, neuronal activation was found to be difficult.^13,14^ Later in 2007, Nicholas Kotov's laboratory reported successful neuronal activation *in vitro* with quantum dots. In this work, quantum dots were embedded into a thin film by layer-by-layer assembly.^15^ In 2010, Arnd Pralle's laboratory reported successful *in vitro* neurostimulation in a magnetic field with magnetic nanoparticles dispersed in water, triggering action potentials in neurons by heating temperature-sensitive ion channels.^16,17^ The need for a thin film was eliminated in this work. Later, water-dispersed gold nanoparticles were also shown to activate neurons *in vitro* in an optical field.^18^

**FIG. 2.**
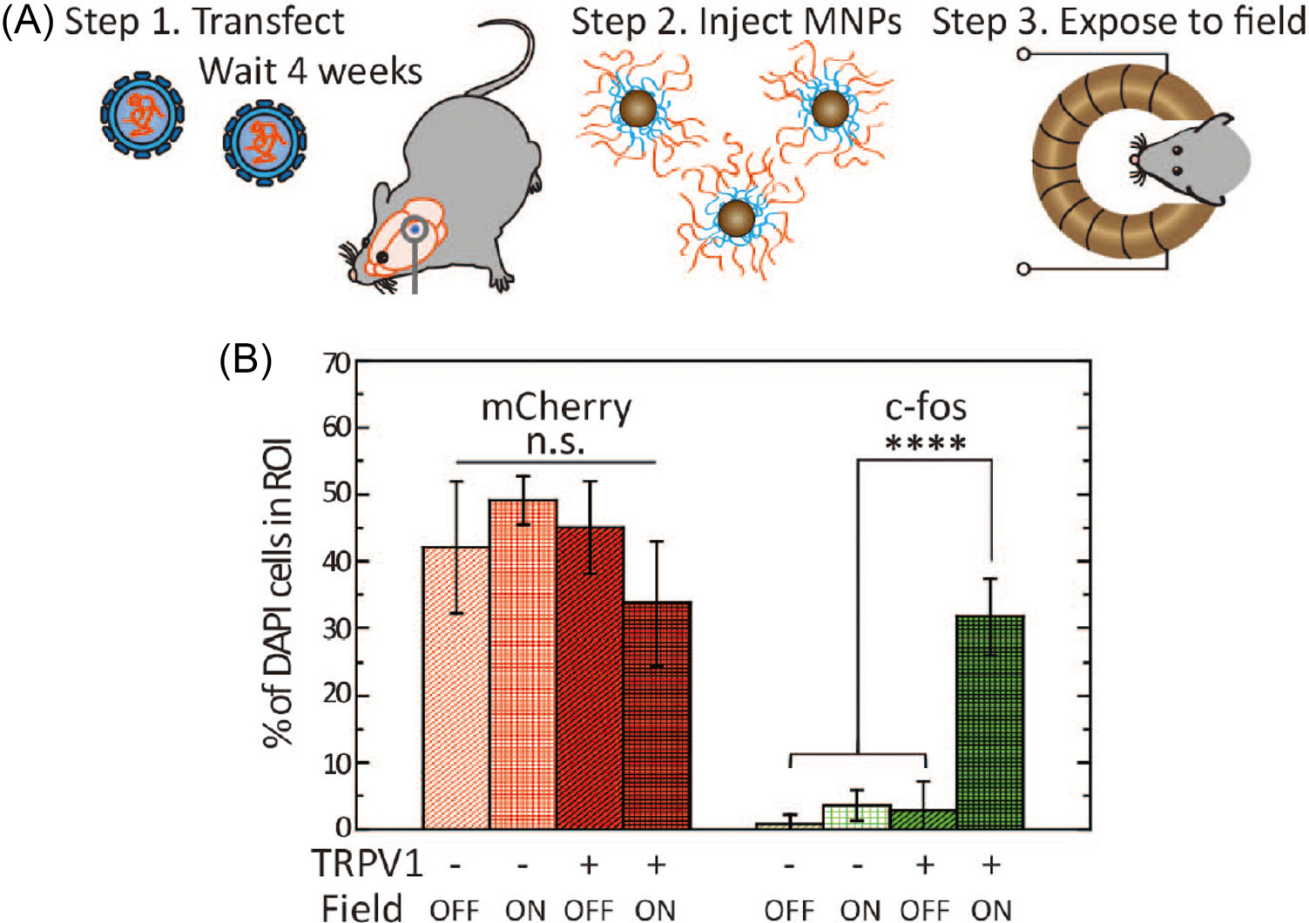
First successful demonstration of NNSP in live animals. A magnetic stimulator was placed on the mouse skull to generate a magnetic field. Magnetic nanoparticles were used in the brains of living mice to increase the expression of c-fos protein in brain cells. (a) The experimental procedure used. (b) Measurement results of c-fos protein expression of brain cells in living mice. Adapted with permission from Chen *et al.*, Science 347, 1477–1480 (2015). Copy right 2015 AAAS.

In 2015, Polina Anikeeva's laboratory published the first NNSP in living animals.^2^ In this work, magnetic nanoparticles were used in the brains of living mice and the expression of c-fos protein in brain cells was enhanced by remote control with a magnetic stimulator placed outside the animal bodies (Fig. 2). Since Anikeeva's groundbreaking publication, several other research groups have reported *in vivo* NNSP, with the reported neurological changes ranging from feeding stimulation,^19^ memory recall,^20^ to near-infrared image vision.^21^

## III. BARRIERS TO CLINICAL TRANSLATION

The wide range of physiological responses achieved in the above animal experiments by different research laboratories strongly indicates the potential of NNSP for medical purposes. However, to realize this vision, great challenges in clinical translation exist and need to be overcome. We outline the major barriers as follows:

(1) Brain delivery of nanomaterials (the “delivery problem”): The above-mentioned animal studies used highly invasive methods to deliver nanoparticles to the sites of interfacing with the nerve cells. The delivery typically involves surgical implant or direct injection into the brains through the skulls. Highly invasive delivery to the nervous system could cause poor patient compliance, create serious side effects to patients, and in some cases be simply impractical to perform.
(2) Safety of the nanomaterials: A number of issues, such as toxicity, biodistribution, immune response, elimination, and undesired responses to external field, need to be comprehensively examined.^22^ Bio-safety data are particularly lacking for nanomaterials in brains. In designing a nanomaterial-based neurostimulation technology, both efficacy and safety information need to be taken into consideration; in many cases, compromises must be made.
(3) Safety of the neurostimulation device: To be used on patients, the neurostimulation device needs regulatory approval to ensure its safety. The above-mentioned animal studies typically used devices that have not been approved for clinical applications.^2,19–21^
(4) Safety of the genetic modification treatments: Genetic modifications to nerve cells are often used in the above-mentioned animal studies of NNSP, in order to specifically label a selective group of cells with nanoparticles.^2,19–21^ These treatments are highly difficult to receive regulatory approval. This is because, when an organism is modified at the genetic level, the changes are fundamental and any undesired change could have powerful impact on the organism's life, e.g., leading to cancers.
(5) Cross-species differences: The biological systems and processes in different species can have significant differences. Thus, the experimental results obtained using one animal species cannot be directly translated to another animal species (including human). Cross-species differences in the inner workings of organisms need to be taken into consideration for the translation. Specifically, comprehensive information on cross-species differences in bio-transport, neural circuits, and bio-safety is needed for clinical translation of NNSP.

## IV. TACKLING THE DELIVERY PROBLEM IN CLINICAL TRANSLATION

**FIG. 3.**
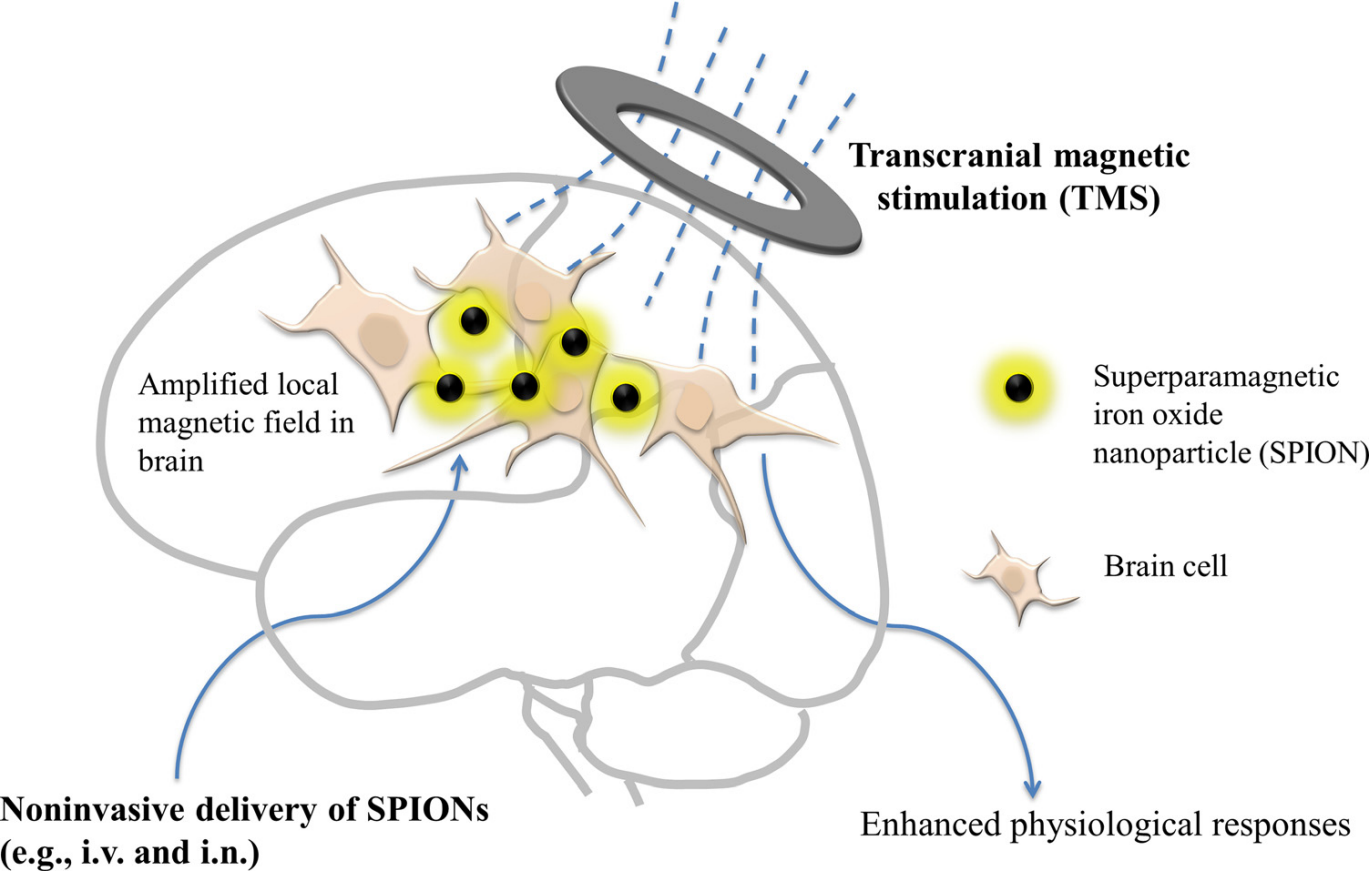
nSPION-TMS technology platform for clinical translation of NNSP. i.v.: intravenous injection. i.n.: intranasal administration.

Each of these barriers is formidable; all of them need to be overcome for eventual clinical applications. Recently, in collaboration with a neuromedicine group, we made a preliminary attempt to tackle the barriers in clinical translation of NNSP.^23^ We introduce a technology platform dubbed nSPION-TMS (noninvasively delivered superparamagnetic iron oxide nanoparticles-based transcranial magnetic stimulation) for medical applications of nanomaterial-based brain stimulation (Fig. 3). In this technology platform, we focus on tackling the delivery problem, while also taking the other translational barriers into consideration.

In nSPION-TMS, TMS is responsible for generating a magnetic field in the brains; it is a clinically approved noninvasive brain stimulation method.^24,25^ Furthermore, nanoparticles are noninvasively delivered into brains, avoiding the use of highly invasive brain delivery methods such as surgical implants and direct injection into brains through skulls. In clinical practice, “noninvasive brain delivery” could include intravenous injection (i.v.) and intranasal administration (i.n.). A large body of literature has been accumulated in developing ways to overcome the blood−brain barrier (BBB), so that exogenous matters (e.g., nanomaterials and therapeutic proteins) can be introduced intro brains in a noninvasive manner.^26^ Both intravenous injection and intranasal administration have been shown to deliver significant amount of nanomaterials into brains, when advanced techniques are used to facilitate nanomaterials' crossing of BBB.^26^ However, prior to our recent paper,^23^ it was unclear whether noninvasive brain delivery of nanomaterials was feasible for NNSP. In our paper, we combined two techniques to facilitate nanoparticles' BBB crossing: (1) coating nanoparticles with chitosan, a natural polymer facilitating BBB crossing (i.e., a chemical method). Chitosan could improve BBB crossing by increasing bioadhesion and opening intercellular tight junctions.^26^ (2) Using a permanent magnet on top of the skull to attract magnetic nanoparticles with a magnetic force (i.e., a physical method). Because the nanoparticles possessed superparamagnetism, they became magnetic in the magnetic field produced by the permanent magnet. As a result, an attraction magnetic force was generated between the permanent magnet and the nanoparticles. This attraction force was used to facilitate BBB crossing and to guide the movement of nanoparticles in the brains. Using healthy rats as the animal models, a key discovery in our paper was that the magnetic nanoparticles delivered into brains using the advanced noninvasive delivery techniques were sufficient to generate significant physiological responses, combined with a brain stimulation device to generate the magnetic field. The physiological responses reported in the paper included increased motor evoked potential and enhanced c-fos protein expression. This finding suggests that the delivery problem is a solvable one. The logical next step is to further improve noninvasive brain delivery ability, and to conduct brain stimulation experiments on diseased animal models to yield a medically relevant outcome.

In principle, the treatment of a variety of neurological diseases, e.g., Alzheimer's disease, Parkinson's disease (PD), depression, stroke, and epilepsy, could benefit from the nSPION-TMS technology. Take PD as an example, TMS has already shown medical benefits in many clinical trials to treat PD;^27,28^ thus, delivering SPIONs to the brain parenchyma region to be stimulated by TMS (e.g., primary motor cortex, M1) could amplify the magnetic field in that region,^23^ enhance the physiological responses,^23^ and produce medical benefits such as improved motor function recovery for the patients (or PD animal models), compared with using TMS alone. Furthermore, the combined use of noninvasive brain delivery of SPIONs and the noninvasive brain stimulation method TMS makes nSPION-TMS a noninvasive treatment method, which is highly desirable for PD patients.

In addition to tackling the delivery problem, the nSPION-TMS technology platform also addresses several other barriers in clinical translation: (1) the nanomaterials used (SPIONs) are based on iron oxide nanoparticles, which have been clinically approved for uses in treating iron deficiency and as medical imaging probes.^29^ In addition, in our recent paper, a synthesis method was developed to produce chitosan-coated SPIONs without introducing toxic organic small molecule ligands.^23^ (2) As mentioned above, the brain stimulation device used is TMS, which has been clinically approved to treat depression.^24,25^ In the brain tissues, SPIONs can amplify the magnetic field of TMS, thereby enhancing brain stimulation effects.^23^ This ability of SPIONs can mitigate key limitations of conventional TMS, namely lack of stimulation depth and intensity in the brain.^24,25^ (3) In the nSPION-TMS design, genetic modification to brain cells is not necessary. In the case that specific stimulation of a group of brain cells with SPIONs is needed, in principle a recognition molecule (e.g., antibody, aptamer, and nanobody) could be chemically conjugated to the SPION surface.^18^ It is worth noting that in our recent paper this has not been experimentally achieved. Finally, cross-species differences are not addressed in this work, and need to be studied in future work using large animal models. For example, studies are needed to investigate the biological transport of nanoparticles in large animal models, and to develop the brain delivery methods accordingly (e.g., tailored magnet design). Additionally, the cross-species differences in biological responses to neurostimulation need to be examined.

## V. CONCLUDING REMARKS

The brain is today's frontier. In addition to understanding the complex and intricate workings of the brain, a grand challenge is to provide real help to patients of neurological diseases, most of which have no cure today. Treating these diseases is in need of innovational strategies such as NNSP and tissue engineering. In this paper, we have outlined the major barriers in the clinical translation of NNSP. We have introduced a technology platform nSPION-TMS to address these barriers. We have discussed our published experimental results with nSPION-TMS and the limitations of these results. It is our hope that nSPION-TMS would inspire more ideas and efforts in the scientific community to tackle the translational challenges of NNSP.

## AUTHORS' CONTRIBUTIONS

Z.X. and J.X. contributed equally to this work.

## Data Availability

Data sharing is not applicable to this article as no new data were created or analyzed in this study.
